# Impact of COVID-19 Pandemic on Academic Stress and Perceived Classroom Climate in Spanish University Students

**DOI:** 10.3390/ijerph19074398

**Published:** 2022-04-06

**Authors:** Nicolás Ruiz-Robledillo, Juan Vela-Bermejo, Violeta Clement-Carbonell, Rosario Ferrer-Cascales, Cristian Alcocer-Bruno, Natalia Albaladejo-Blázquez

**Affiliations:** 1 Department of Health Psychology, University of Alicante, 03690 Alicante, Spain; nicolas.ruiz@ua.es (N.R.-R.); rosario.ferrer@ua.es (R.F.-C.); cristian.albru@ua.es (C.A.-B.); natalia.albaladejo@ua.es (N.A.-B.); 2 Department of Innovation and Didactic Training, University of Alicante, 03690 Alicante, Spain

**Keywords:** COVID-19, academic stress, perceived classroom climate, university, students

## Abstract

The COVID-19 pandemic has caused several changes in society, especially in the educational context, where several learning methodologies and social interactions have been modified significantly. This fact could have had a negative impact on academic stress levels of students and the classroom climate, especially in the university context. The main aim of the present study was to identify changes in academic stress and the perceived classroom climate caused by COVID-19 in a sample of Spanish university students. Academic stress was evaluated trough the Stressor Academic Scale (SAS) and perceived classroom climate employing the Perceived Classroom Responsibility Climate (PCRC) questionnaire. A longitudinal study was conducted. 135 students (97 females and 38 males) from the Gastronomy (*n* = 31) and Criminology (*n* = 104) degrees were evaluated before and after the COVID-19 lockdown in Spain. Academic stress levels and perceived classroom climate were analyzed before (Time 1) and after (Time 2) the lockdown declaration. An increase in academic stress was found, especially in the categories regarding Teachers’ Methodological Deficiencies, Academic Over-Burden and Beliefs About Performances. Females and final year students suffered higher levels of academic stress. No differences were found between Time 1 and 2 in perceived classroom climate. The obtained results point out a significant increase of academic stress in university students due to the COVID-19 pandemic in Spain. The implemented educational changes and the uncertainty that resulted from the pandemic could have a significant negative impact on mental health in this population, resulting in higher levels of academic stress, especially in females and final year students. Future studies should analyze the strategies that students are employing to cope with these educational challenges and intervention strategies to promote them in the context of higher education.

## 1. Introduction

### 1.1. Academic Stress

Stress is currently one of the most recurrent pathologies in the population, associated with states such as nervousness, tension, anxiety, fatigue or depression, and also generated by contexts such as school or work pressure [1,2]. In the particular case of academic stress, this framework of abundant tension appears from preschool periods to university life, a space in which learning intervals are altered by the demands imposed in the various educational cycles [3]. In addition, there is a set of recurrent psychosocial stressors in this area, where concerns about academic performance, the management of exams, the type of relationship with peers and teachers, and the teacher’s methodology or workload stand out [4]. In short, this type of stress is framed in a systemic and psychological process in which students, under the condition of high academic demand, are affected by a series of symptoms that generate an evident cognitive and physical imbalance [3,5].

If we take a look at the literature focusing on the variables in academic life that generate stress or anxiety, there are three main groups that cause this type of ailment in university students: aspects linked to assessment processes; situations marked by overwork; and variants within the teaching-learning process such as the links between students and the teaching staff (or within the student groups themselves), organizational components (timetables, syllabuses, overlapping of subjects), or teaching methodologies [6,7].

The evaluation processes linked to the types of tests and exams, the management of their content and time for completion and, especially, their direct relationship with the options of higher education or job opportunities, will generate a considerable amount of stress on students who directly equate their academic and personal success or failure to the passing of these assessment tests [5,6,8].

In direct relation to the previous stressor, it is worth highlighting the work overload in terms of the administration of exams in short periods of time, uncertainty regarding the teacher’s assessment method, and the high levels of demands [9,10]. Other noteworthy aspects in this consideration of overwork as a stress factor would be the absence of free time, the difficulty in reconciling academic and personal life or the high load of autonomous work outside the classroom sessions (study hours, internships and research work in other areas outside the academic environment, etc.) [11].

With regard to the organizational structure of the curricula, several lines of research have established particular stressor variables such as the relevance of the clarity of the educational programs or the impact of the recent changes in teaching methodologies in accordance with the approaches of the European higher education area [12,13]. Other relevant issues would be stress indicators in direct relation to particular degrees or to the follow-up of subjects based on lecture-based or innovative methodologies [14].

### 1.2. The Moderating Effects of Sex and the Year of Study on Academic Stress

Based on the results of previous research, the effects of academic stress are mediated by socio-demographic and socio-educational factors; in addition, two fundamental variables deserve special mention: the students’ sex and the particular academic year that they are in [15,16,17,18,19,20]. Women and men share many environmental influences; however, they are socialized according to different patterns that can affect the way they face stressful experiences and the modes in which they generate specific responses to alleviate these stressful situations [15]. Regarding sex, studies on the subject insist on its establishment as a variable that has a significant impact on the gestation of stress in school contexts. The general data corroborate an irrefutable fact: higher levels of stress and anxiety are found among the female population compared to the male population [16]. This reactivity and vulnerability to stress among women is directly related to their greater concern for pleasing adults (e.g., parents and teachers) and their way of dealing with the evaluation process [16]. In the academic field, the fundamental problem is the type of coping strategies used by men and women. The existing literature affirms that women tend to resort mostly to social supports to combat stress, while men seem to be more willing to establish strategies such as carrying out some type of action that involves solving the problem, rejecting it or modifying it through alternative activities [17]. Likewise, new studies are needed to identify possible sex differences during special stressful situations such as that presented by the COVID-19 pandemic.

On the other hand, stress is perceived differently depending on the academic year, with an increase in the appearance of symptoms in the first years and a decreasing in their appearance in the last year of their degree [18]. The existing literature, especially linked to the field of health sciences, establishes that first-year students start their academic career with high levels of anxiety due to factors such as fear of the unknown, high expectations that are wrongly set (for example, in relation to future salaries after completion of the degree) or to the need to leave home and settle in a new space [18,19]. These stress levels tend to decrease for a prolonged period of time in the last semesters of the degree program, although an upturn can be seen in the months prior to graduation when the student becomes aware of the future responsibility of practicing the profession, the need to continue with pre-doctoral studies or the extreme competitiveness of the labor market [20]. Although these findings refer to medical students [20], these results could be generalized to university students in general, as the uncertainty and labor market integration could be the most common stressors in this population.

### 1.3. Perceived Classroom Climate

Another noteworthy aspect would be the multidimensional variable known as classroom climate and its link with stress, i.e., how spatial factors (physical environment, furniture) and personal issues (relationships between teachers and students or among students themselves), together with teaching methodology, influence the emotional and attitudinal aspects of university groups [21]. On the one hand, the classroom must be an organized space with adequate conditions (for example, in the case of chairs or tables), with technological and didactic equipment conducive to a correct and dynamic teaching performance [21]. On the other hand, the teaching staff must possess competencies such as the mastery of the contents, the management of an evaluation system based on justice and the correct measurement of learning or a dynamic and updated teaching methodology linked to a respectful interaction with the students [22]. Finally, the relationship between the actors in the teaching and learning process will be based on understanding and fairness in order to establish a positive climate of trust, respect, affection and kindness [23].

### 1.4. The Impact of COVID-19 on University Context

On 11 March 2020, the World Health Organization (WHO) declared the COVID-19 virus to be a global pandemic. The consequences of this problem have greatly affected health, the economy and education; in the latter area, the stress generated in the academic, teaching and family community has been evident given the uncertainty generated by the suppression of face-to-face classes, the fear of contagion and the implementation of a distance learning methodology [24]. In addition to the aforementioned sources of stress, and specifically in the university environment, this new social reality has introduced new stressors such as the irrational fear of contagion, social distancing that leads to isolation and distrust, together with the loss of positive attitudes such as security, predisposition, or effort [25,26]. Similarly, distance learning has led to various problems such as integration difficulties due to not knowing their classmates and teachers face-to-face, especially in the case of first year students, and the limitations on experimentation in the social side of university life [27]. It would be necessary to add other problematic issues related to the impossibility of having adequate equipment to follow online sessions or the lack of self-discipline or skills to carry out virtual learning activities [28]. In short, the sum of all these factors can lead to an increasingly stressful environment that generates uncertainty, anxiety, depression, loss of motivation and, consequently, often leads to school dropout [29,30,31,32,33].

### 1.5. Aims of the Study

To sum up, academic activities managed within the university framework represent for many students a worrying source of stress given the high academic, social and personal demands. Although there is a considerable and recent body of research on academic stress, few studies have focused on its impact on university students and, specifically, in the current pandemic context. Since the beginning of 2020, students have been facing a new academic scenario that has abruptly changed the teaching reality [32,33]. During this process they have been confronted with problems and difficulties such as limitations in terms of accessibility and connectivity to the Internet, the unfavorable perception of didactics, changes in teaching strategies and methodologies of professors, as well as work overload and continuous exposure to computers, laptops and smartphones [29,30,31,32,33]. Therefore, the main objective of this study is to analyze the possible changes in academic stress and perceived classroom climate scores before and after COVID-19 lockdown in a sample of Spanish university students. Furthermore, the study aimed to identify differences in the possible changes in academic stress and perceived classroom climate, considering the sex of students and the year of study in which they were enrolled.

## 2. Materials and Methods

### 2.1. Research Method and Design

The present study was based on an observational research method, following a longitudinal design.

### 2.2. Research Context and Participants

The present study was conducted at the University of Alicante, located in the province of Alicante in Spain. This public university has an enrollment of more than 25,000 students every year, and includes several faculties with different degrees, including graduate programs. Participants of the study were students from this university enrolled in the Gastronomy Criminology degree programs. During the study development, those in the Gastronomy degree program included 75 students, and the Criminology degree program included 249 students. Students from these degrees were chosen to participate in the study because they belong to different areas of knowledge, with diverse teaching methodologies. Although the number of students enrolled in these degrees is relatively small, the obtained sample could be representative of the diversity of students at the university. Furthermore, in the research, students from 1st, 3rd and 4th course participated. As it has been previously explained, different stressors could affect students depending on the year of study, and hence, the impact of COVID-19 could be different if students are enrolled in the initial year (1st), in an intermediate year (3rd) or at the final year of the degree (4th). Sociodemographic characteristics of the sample are shown in Table 1.

### 2.3. Instruments

#### 2.3.1. Sociodemographic and Educational Data

An elaborated ad-hoc questionnaire was employed to evaluate the sociodemographic and educational data of participants. Age, sex, employment status, monthly income, relationship status, current studies, and the current year of study were evaluated.

#### 2.3.2. Academic Stress

The Stressor Academic Scale (SAS) was used to analyze levels of academic stress in participants [4]. This scale includes 54 items with a 5-point Likert-type rating scale (1 = never; 2 = sometimes; 3 = quite often; 4 = almost always; 5 = always) structured around eight categories: teachers’ methodological deficiencies; academic over-burden; beliefs about performance; public interventions; negative social environment; exams; content worthlessness; and participation difficulties. The scale was shown to be highly reliable in each subscale, with a Cronbach’s alpha ranging between 0.81 and 0.94, and for the total scale α = 0.96 [4].

#### 2.3.3. Classroom Climate

Perceived Classroom Climate was evaluated employing the questionnaire developed by Fernández-Río and colleagues [34]. The Perceived Classroom Responsibility Climate Questionnaire (PCRC) is composed of 10 items ranked on a 7-point Likert scale ranging from strongly disagree (1) to strongly agree (7). These 10 items could be grouped around two specific factors: the responsibility climate generated by classmates and the responsibility climate generated by the teacher. Higher scores are indicative of a positive classroom climate. This instrument has demonstrated adequate psychometric properties, with a Cronbach’s alpha > 0.80 in both subscales [34].

### 2.4. Procedure

**Figure 1:**
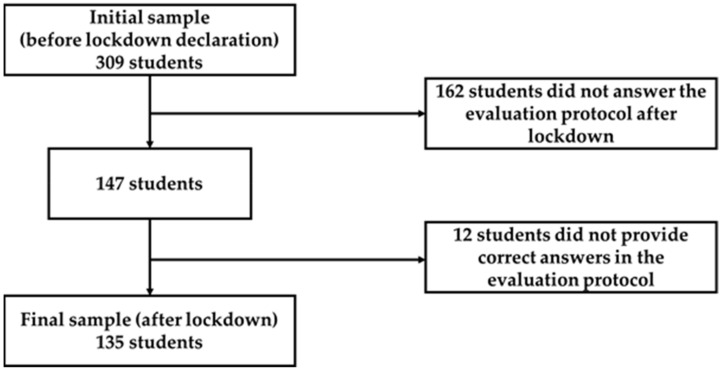
Flow-diagram of participant recruitment.

The recruitment procedure was based on convenience sampling. During the first part of the research, before the lockdown declaration due to COVID-19, students were contacted in class, and the researchers explained the aims of the study. The researchers indicated that participation was completely confidential, anonymous, and voluntary, and that their participation did not influence course grades in any way. Students who agreed to participate in the study were sent an email with the link to the evaluation protocol configured on the Google Form platform. To protect the confidentiality and anonymity of the data, codes were assigned to identify the participants following a pseudonymization process. These anonymized codes would allow for the linking of the initial responses (Time 1) to the subsequent ones (Time 2). The first evaluation was completed by students during January 2020, and 309 answered the questionnaires (which entails a 95.37% of response rate). Then, on 14 March 2020, the state of alarm due to COVID-19 started in Spain and then ended on 21 June 2020. Following the declaration of the state of alarm, all face-to-face classes in Spanish universities were cancelled. This fact resulted in a significant change in teaching methodologies and the lack of social interaction between teachers and students and between students themselves. Furthermore, the declaration of the state of alarm led to house confinement, which significantly affected students’ personal, academic, and social lifestyles. When state of alarm was finished, an email was sent to the students who agreed to participate at the beginning of the study including a new link with an evaluation protocol in Google Forms. From the initial sample, 147 students responded to the questionnaires (a response rate of 47.57% from respondents of Time 1), but the final sample was composed of 135 participants because 12 students did not provide correct answers in the evaluation protocol, mainly due to incomplete responses in the questionnaire (several items without responses) or an entirely unfilled questionnaire (see Figure 1).

Only individuals who agreed to participate and signed the informed consent were included in the study. To protect the confidentiality and anonymity of the data, codes were assigned to identify the participants. The email addresses of students were only used to send them the links to the study. Emails or any personal information did not appear in the results derived from the Google Forms. The research was conducted following the guidelines of the Declaration of Helsinki and the European Union Good Clinical Practice Standards, and the study was approved by the Ethical Committee of the University of Alicante (UA-2020-11-20).

### 2.5. Data Analysis

Descriptive and frequency analyses were performed for sociodemographic and educational data. To analyze differences in academic stress and classroom climate before and after the lockdown declaration due to COVID-19 in students, a paired samples *t*-test was performed. Repeated Measures ANOVA analysis was employed to identify possible differences in academic stress and the classroom climate before and after the lockdown including the factors “sex” and “year of course” as between-subject factors. Bonferroni correction was applied in post-hoc analysis. All statistical analyses were performed using JAMOVI version 1.6.23, considering any *p* < 0.05 as significant.

## 3. Results

### 3.1. Scores in Academic Stress and Perceived Classroom Climate in Time 1 (before Lockdown Declaration) and Time 2 (after Lockdown)

Table 2 shows the scores obtained by participants in each subscale of academic stress and the perceived classroom climate in both times of evaluation (before and after lockdown). Moreover, the percentage of change from Time 1 to Time 2 has been included.

### 3.2. Differences in Academic Stress and Perceived Classroom Climate in Time 1 (before Lockdown Declaration) and Time 2 (after Lockdown)

With the aim of analyzing the possible differences between Time 1 and Time 2 in academic stress and perceived classroom climate, Paired sample t-tests were performed. As can be observed, differences were found in TMD (Teachers’ Methodological Deficiencies), AOB (Academic Over-Burden), BAP (Beliefs About Performances) and TAS (Total Academic Stress). In all cases, higher scores were found in Time 2 in comparison to Time 1 (for all, *p* < 0.05) (Table 3).

### 3.3. Differences in Academic Stress in Time 1 before Lockdown Declaration) and Time 2 (after Lockdown) by Sex and Year of Study

No significant interaction effect “Time*Sex” was found in any of the dimensions of academic stress. However, a main effect of “Sex” was found in TMD F(1,133) = 13.7, *p* < 0.001, η^2^ = 0.077; AOB F(1,133) = 16, *p* < 0.001, η^2^ = 0.087; BAP F(1,133) = 9.95, *p* = 0.002, η^2^ = 0.061; PI F(1,133) = 13.6, *p* < 0.001, η^2^ = 0.082; NSE F(1,133) = 5.56, *p* < 0.020, η^2^ = 0.032; EX F(1,133) = 34.6, *p* < 0.001, η^2^ = 0.184; PD F(1,133) = 7.46, *p* = 0.007, η^2^ = 0.040; and TAS F(1,133) = 22.1, *p* < 0.001, η^2^ = 0.125. In all cases, except in the case of Content Worthlessness, female students showed higher stress levels than males (Table 4).

A main effect of the factor “Year of study” was found in AOB F(2,132) = 6.57, *p* = 0.002, η^2^ = 0.073; NSE F(2,132) = 15.5, *p* < 0.001, η^2^ = 0.153; CW F(2,132) = 5.08, *p* = 0.008, η^2^ = 0.054; PD F(2,132) = 5.51, *p* = 0.005, η^2^ = 0.058; and TAS F(2,132) = 3.67, *p* = 0.028, η^2^ = 0.046. In the case of AOB, post hoc analyses revealed differences between students of first year in comparison to students of the third (*p*_bonferroni_ = 0.034) and fourth (*p*_bonferroni_ = 0.001) years. First year students showed lower levels in the AOB dimension in comparison to the other groups. Regarding NSE, differences were found between all groups. In this sense, students from the first year showed lower levels of NSE than students of the third (*p*_bonferroni_ = 0.011) and fourth years (*p*_bonferroni_ < 0.001). Moreover, students in their third year exhibited lower scores in NSE than the students in their fourth years (*p*_bonferroni_ = 0.003). Differences in CW were found between students in their first year and students in their fourth (*p*_bonferroni_ = 0.005), showing that the former had the lower scores. In the case of PD, students of the first year exhibited lower levels than students of the fourth year (*p*_bonferroni_ = 0.004). For TAS, differences were found between students in their first year and students in their fourth year (*p*_bonferroni_ = 0.028), showing that the former had the lower scores (Table 5). No significant interaction effect “Time*Year of study” was found in any of the dimensions of academic stress.

### 3.4. Differences in Perceived Classroom Climate in Time 1 before Lockdown Declaration) and Time 2 (after Lockdown) by Sex and Year of Study

Regarding Classroom climate, no significant effect of the interaction “Time*Sex” or a main effect of “Sex” was found (Table 6).

However, a significant effect of the interaction “Time*Year of study” was found in RCC F(2,132) = 4.124, *p* = 0.018, η^2^ = 0.019 and in RCT F(2,132) = 3.594, *p* = 0.030, η^2^ = 0.015. Post hoc analyses revealed significant differences between groups of students in scores on RCC in Time 1 (before lockdown declaration). In this sense, students from their first year showed higher scores than students from their fourth year (*p*_bonferroni_ < 0.001). The same results were found for Time 2 (*p*_bonferroni_ = 0.018). However, in the case of RCT, post hoc analyses did not reveal significant differences (*p* < 0.05) (Table 7). Furthermore, a main effect of the factor “Year of study” was found for RCC F(2,132) = 6.95, *p* = 0.001, η^2^ = 0.064. Post hoc analyses showed that students in their first year obtained higher scores than students from the fourth year in this dimension (*p*_bonferroni_ < 0.001) (Table 7).

## 4. Discussion

The COVID-19 crisis led to the closure of universities and forced students not only to change their general living conditions, but also to substantially adjust their daily academic work, long-term projects and expectations. Therefore, the main objective of this article was to address the effects of COVID-19 on the academic stress levels and perceived class-room climate of university students, thus observing their vulnerability or academic resilience in a sample analysis of students from the University of Alicante (Spain).

The results show higher scores on stress perceived by students at Time 2 compared to Time 1 in the majority of evaluated dimensions. Specifically, the dimensions of teacher’s methodological deficiencies, academic over-burden, beliefs about performance, and total academic stress show statistically significant differences between Time 1 and Time 2 in favor of the latter. These results are in line with previous studies showing elevated rates of academic stress during the pandemic, concern about the possible decrease in their academic performance, as well as discomfort with the use of technologies in teaching [25]. In addition, during the pandemic, students reported higher rates of psychological problems such as anxiety and depression, which, together with increased academic stress, can lead to general mental health problems in this group [35,36]. In this sense, the results show that the variables that generated the most stress in the students during the confinement were the methodological deficiencies presented by the teachers, probably due to the urgency with which they had to adapt to an online teaching system, as well as the academic over-burden that this produced in the students and the greater concern about their academic performance in this new and uncertain situation derived from the pandemic [37]. In fact, it can be deduced from previous studies that, in general, students are stressed by this type of situation, and these high levels of stress have been related to the changes in the educational dynamics of the pandemic context, including, for example, difficulties in carrying out group work, new dynamics of online exposure, or new types of evaluation, among others [38,39]. Previous work by different researchers has shown that psychological distress was mainly related to several stressors, as perceived by the participants, which is in line with our results and include the following: academic future, task overload, worsening interpersonal conflicts, and restrictions in pleasant social contact; and not so much to aspects related to the spread of the disease and its consequences for physical health [35,36]. However, contrary to expectations, no differences were found in several dimensions of academic stress. Although the majority of research has evaluated the negative consequences of COVID-19 on academic stress levels, some studies have analyzed the possible protective factors which could buffer the negative consequences of the pandemic situation. In this regard, the adaptability of students has shown to be a protective factor in this regard [40,41]. Adaptability could be conceptualized as the ability of students to make efficient adjustments in the cognitive, behavioral and emotional domains in response to ambiguous situations [40,41]. As has been demonstrated, higher levels of adaptability in students has been related to lower levels of anxiety and hopelessness during the COVID-19 pandemic. Despite the fact that these variables were not evaluated in this study, their effects on the participants of our study could be a plausible explanation of some of the obtained results, especially in the case of non-significant differences between Time 1 and 2.

Regarding gender differences, our results show differences in the majority of the evaluated dimensions of academic stress, with women showing higher scores. Specifically, significant differences were found in the methodological deficiencies of teachers, academic over-burden, beliefs about performance, public speaking, negative social environment, exams, participation difficulties and total academic stress. These differences are consistent with the results obtained in previous studies showing higher levels of academic stress in female students [42,43,44]. These studies revealed that stress and anxiety perceived by university students increased across the board following the emergence of COVID-19, and that women reported worse well-being compared to men [42,43,44]. On the other hand, our results are in agreement with other research assessing the level of anxiety and the general different perceptions of stress between men and women, the latter being more likely to suffer anxiety as well as the physical, psychological and behavioral reactions it entails [45,46].

This evidence leads to the need to consider the explanatory mechanisms underlying this phenomenon. Among them, we find the sociocultural perspective of stress, which assumes that stressors may be similar for men and women, but not in the way they are valued, how they are reacted to, how they affect the individual and how they are dealt with as a consequence of the unequal education for men and women [17]. In this sense, the former are encouraged to be independent, while women are encouraged to seek social support as the main response mechanism to problematic situations, something really difficult during a lockdown situation [17,47].

On the other hand, coping strategies play a central role in the management of stress in a different way between men and women. In general, men tend to resort to the use of active coping strategies (mainly cognitive and physical activity), while women focus on social strategies of support-seeking [17,43]. Likewise, other studies have reported that anxiety and depressive symptoms were strongly related to coping strategies, especially when these were avoidance strategies (which were more frequently used by women [48]). Other studies point to significant differences between fear of academic failure and the online and home environment among male and female students [29].

The results of this research show statistically significant differences in the perception of academic stress depending on the academic year, as in previous studies [15,39,49]. Specifically, first-year students showed lower levels of academic over-burden compared to the rest of the students in other courses. The effects of a negative social environment were also lower in first-year students compared to third and fourth-year students, with senior students showing the highest scores on this dimension. As for the content dimension, first-year students had significantly lower scores compared to fourth-year students. Likewise, fourth-year students perceived greater difficulties in participation compared to first-year students. These results can be explained by the fact that first-year students had not had time to establish an extensive network of social relationships due to confinement compared to students in higher grades who already had previous experience in this regard [50]. Among the stressors related to the teaching-learning process, special relevance has been given to the role played by the social relationships established among the individuals who form part of the group and especially those of the students among themselves [39]. This fact has demonstrated to be especially important in university students, as it has been demonstrated to be one of the main worries of the first-year university students [51]. In this sense, with regard to classroom climate, no significant differences were found according to gender, but significant differences were found according to the academic year studied. Specifically, first-year students obtained significantly higher scores in the Responsibility Climate generated by Classmates dimension compared to fourth-year students. These results are replicated in the data collected both before and after lockdown. This aspect is particularly important and highlights that those students enrolled in advanced years may be at greater academic risk, since it has been shown that a good social climate favors the development and refinement of conflict resolution skills (both personal and social), especially negotiation skills, which produces a stress-reducing effect [39,52]. An adequate social climate in the classroom contributes to the student coping more effectively with the stressful situation or attenuates the experience of stress when it has already occurred [39,53].

Although previous studies have shown that students in the first years of school tend to suffer higher levels of academic stress, our results show that it is the students in the last years who present higher levels of stress when compared to students from first and intermediate years. It is probable that those students have a more critical capacity to assess the negative consequences of methodological changes and uncertainty due to their previous academic experience.

Despite all of this, this work has several limitations. The first is the fact that the evaluations were carried out using an online evaluation platform such as Google Forms. Although it may be an advantage to avoid social contact in times of a pandemic where it has been limited to what is strictly necessary as well as the possibility of contacting a large number of subjects for the sample, the use of online techniques in data collection may result in a high loss of subjects from the sample. On the other hand, the representativeness of the sample is limited to students from two grades at the University of Alicante that, although they are representative of their degree, may present distinctive or particular characteristics with respect to other degrees. In this sense, the small and selective sample is a significant limitation of the study. For this reason, the obtained results should be considered with caution in terms of generalization. Moreover, the fact that students received an email to participate in the research could have conditioned their participation. Since sensitive topics were addressed in the questionnaires, some students maybe held back in their answers. However, the research team explained carefully that the responses were absolutely confidential and no personal and identifying information was to be collected. Nevertheless, to the best of our knowledge, our study is one of the few that has been carried out longitudinally in Spain, which makes the results obtained highly reliable regarding academic stress and the perceived classroom climate during the COVID-19 pandemic.

## 5. Conclusions

One of the tasks of the university as an institution is to assume the challenge of mitigating the impact of academic stress among its students through the articulation of assistance, guidance and socioemotional support processes that facilitate constructive coping with academic demands and minimizing the possible negative effects on their health and general well-being [54,55]. Managing stress, fostering coping resources, promoting motivational support and positive affectivity, training skills to know how to respond to demands and resolve conflicts are actions that institutions should offer to their student community to help students cope effectively with stress, avoid as much as possible their vulnerability to this emotion, and improve their adaptation options and academic performance [54,55]. The results of our study allow the planning of these stress prevention and intervention programs for university students in a more efficient way that is oriented to the characteristics of this group.

In conclusion, the results of our study show a high level of academic stress in university students, especially in women, after the COVID-19 lockdown in Spain. The domains particularly affected by this negative situation were those related to the teacher’s methodological deficiencies, academic over-burden, and beliefs about performance. On the other hand, we observed the negative effect of the pandemic on the academic stress of students and its differential impact depending on the year of study, with fourth-year students being those who, in general, have worse scores in academic stress and a poorer perceived classroom climate.

The COVID-19 crisis caused the closure of universities and forced students not only to change their general living conditions, but also to substantially adjust their daily aca-demic work, long-term projects, and expectations. Therefore, studies such as the present research, which analyzes academic stress factors according to gender and year of study, has practical implications for the educational context. Hence, these are relevant for the design of psychoeducational actions and interventions for the prevention of stress and anxiety that contribute to the improvement of adaptability, well-being, quality of life, and academic performance in university students. In this sense, based on the obtained results, these prevention and intervention educational strategies should take into account the moderating effects of gender and the academic course of students on academic stress, as females and students in their final years seem to be at higher risk to develop negative consequences from stressful situations.

Future studies should analyze the strategies that students use to restore their emotional balance, which could include self-praise, the search for evasive distractions, the enhancement of a sense of humor, the need for professional psychological help, the elaboration of a detailed work plan or even the intensification of religiosity [56,57,58]. This information would allow professionals to develop and implement several educational and psychological strategies to reduce academic stress during this pandemic situation.

## Figures and Tables

**Table 1:** Sociodemographic characteristics of the sample.

Variable	Categories	Total Sample N = 135
Age		21.7 ± 4.39
Sex	Male	38 (28.1%)
	Female	97 (71.9%)
Relationship status	Single	91 (67.4%)
	In a relationship	44 (32.6%)
Employment status	Employed	30 (22.2%)
	Non-employed	105 (77.8%)
Income (monthly)	EUR < 500	115 (87.1%)
	EUR > 500	17 (12.9%)
Current studies	Gastronomy	31 (23%)
	Criminology	104 (77%)
Current year of study	1stcourse	33 (24.4%)
	3rd course	65 (48.1%)
	4th course	37 (27.4%)

**Table 2:** Scores in academic stress and perceived classroom climate in Time 1 (before lockdown declaration) and Time 2 (after lockdown).

	Time 1	Time 2	Percentage Change
**Academic Stress**			
**TMD**	38.4 ± 11.6	41.4 ± 11.9	7.81%
**AOB**	25.2 ± 8.81	27.3 ± 8.37	8.33%
**BAP**	22.31 ± 8.79	24 ± 8.62	7.58%
**PI**	16.48 ± 6.35	16.38 ± 5.87	-0.61%
**NSE**	11 ± 3.84	11.59 ± 4.31	5.36%
**EX**	11.28 ± 4.28	11.30 ± 4.23	0.18%
**CW**	9.57 ± 3.34	10.02 ± 3.70	4.7%
**PD**	6.30 ± 2.59	6.68 ± 2.72	6.03%
**TAS**	140.50 ± 36.48	148.57 ± 35.37	5.74%
**Classroom Climate**			
**RCC**	26.64 ± 5.28	27.01 ± 4.78	1.39%
**RCT**	28.05 ± 6.31	28.42 ± 5.50	1.32%

Teachers’ Methodological Deficiencies (TMD); Academic Over-Burden (AOB); Beliefs About Performances (BAP); Public Interventions (PI); Negative Social Environment (NSE); Exams (EX); Content Worthlessness (CW); Participation Difficulties (PD) and Total Academic Stress (TAS); Responsibility Climate generated by Classmates (RCC) and Responsibility Climate generated by the Teacher (RCT).

**Table 3:** Differences in academic stress and perceived classroom climate in Time 1 (before lockdown declaration) and Time 2 (after lockdown).

	t	df	*p*	Mean Difference	SE Difference	Effect Size (Cohen’s d)
**Academic Stress**						
**TMD**	−3.6431	134	<0.001	−2.9704	0.815	−0.31355
**AOB**	−3.3791	134	<0.001	−2.1407	0.634	−0.29083
**BAP**	−3.0833	134	0.002	−1.6222	0.526	−0.26536
**PI**	0.2659	134	0.791	0.0963	0.362	0.02289
**NSE**	−1.8800	134	0.062	−0.5852	0.311	−0.16180
**EX**	−0.0923	134	0.927	−0.0222	0.241	−0.00794
**CW**	−1.5187	134	0.131	−0.4519	0.298	−0.13071
**PD**	−1.6646	134	0.098	−0.3778	0.227	−0.14327
**TAS**	−3.8514	134	<0.001	−8.0741	2.096	−0.33147
**Classroom Climate**						
**RCC**	−0.751	134	0.454	−0.370	0.493	−0.0646
**RCT**	−0.680	134	0.498	−0.370	0.545	−0.0585

Teachers’ Methodological Deficiencies (TMD); Academic Over-Burden (AOB); Beliefs About Performances (BAP); Public Interventions (PI); Negative Social Environment (NSE); Exams (EX); Content Worthlessness (CW); Participation Difficulties (PD) and Total Academic Stress (TAS); Responsibility Climate generated by Classmates (RCC) and Responsibility Climate generated by the Teacher (RCT).

**Table 4:** Differences in academic stress in Time 1 (before lockdown declaration) and Time 2 (after lockdown) based on Sex.

		Time 1	Time 2	Percentage Change
**Academic Stress**				
**TMD**	Females	40.2 ± 10.9	43.7 ± 11.1	8.71%
	Males	33.9 ± 12.2	35.4 ± 12	4.42%
**AOB**	Females	26.6 ± 8.22	29 ± 7.23	9.02%
	Males	21.4 ± 9.25	23 ± 9.56	7.48%
**BAP**	Females	23.5 ± 8.15	25.5 ± 7.93	8.51%
	Males	19.3 ± 9.78	20.1 ± 9.19	4.15%
**PI**	Females	17.4 ± 6.14	17.7 ± 5.70	1.72%
	Males	14.2 ± 6.39	13.1 ± 5.08	−7.75%
**NSE**	Females	11.5 ± 3.91	12.1 ± 4.49	5.22%
	Males	9.87 ± 3.46	10.4 ± 3.64	5.37%
**EX**	Females	12.4 ± 3.92	12.5 ± 3.82	0.81%
	Males	8.42 ± 3.85	8.34 ± 3.82	−0.95%
**CW**	Females	9.57 ± 2.90	9.96 ± 3.33	4.08%
	Males	9.61 ± 4.33	10.2 ± 4.58	6.14%
**PD**	Females	6.56 ± 2.58	7.09 ± 2.83	8.08%
	Males	5.66 ± 2.56	5.63 ± 2.15	−0.53%
**TAS**	Females	148 ± 32.1	157 ± 29.9	6.08%
	Males	122 ± 40.8	126 ± 38.7	3.28%

Teachers’ Methodological Deficiencies (TMD); Academic Over-Burden (AOB); Beliefs About Performances (BAP); Public Interventions (PI); Negative Social Environment (NSE); Exams (EX); Content Worthlessness (CW); Participation Difficulties (PD) and Total Academic Stress (TAS).

**Table 5:** Differences in academic stress in Time 1 (before lockdown declaration) and Time 2 (after lockdown) based on Year of study.

		Time 1	Time 2	Percentage Change
**Academic Stress**				
**TMD**	1 year	36.1 ± 12	40.5 ± 12	12.19%
	3 year	38.3 ± 11	41.7 ± 12.5	8.88%
	4 year	40.6 ± 12.1	41.6 ± 10.8	2.46%
**AOB**	1 year	21.5 ± 7.8	23.5 ± 7.46	9.3%
	3 year	25.8 ± 8.67	27.3 ± 7.62	5.81%
	4 year	27.2 ± 9.16	30.6 ± 9.15	12.5%
**BAP**	1 year	21.6 ± 10.2	22.9 ± 8.81	6.02%
	3 year	22.4 ± 7.99	23.6 ± 7.75	5.36%
	4 year	22.7 ± 9.06	25.4 ± 9.88	11.89%
**PI**	1 year	15 ± 5.8	14.7 ± 5.43	−2%
	3 year	17.4 ± 6.88	17.1 ± 6.35	−1.72%
	4 year	16.2 ± 5.75	16.7 ± 5.22	3.09%
**NSE**	1 year	8.88 ± 2.32	9.27 ± 3.05	4.39%
	3 year	10.7 ± 3.79	11.7 ± 4.20	9.35%
	4 year	13.5 ± 3.72	13.5 ± 4.60	0%
**EX**	1 year	10.5 ± 4.53	11.1 ± 4.64	5.71%
	3 year	11.4 ± 3.93	11.4 ± 4.19	0%
	4 year	11.8 ± 4.65	11.3 ± 4.06	−4.24%
**CW**	1 year	8.06 ± 3.34	8.82 ± 3.85	9.43%
	3 year	10.3 ± 3.53	10.7 ± 3.97	3.88%
	4 year	9.68 ± 2.58	10 ± 2.82	3.31%
**PD**	1 year	5.58 ± 2.45	5.64 ± 2.12	1.08%
	3 year	6.17 ± 2.42	6.71 ± 2.69	8.75%
	4 year	7.19 ± 2.83	7.57 ± 3.01	5.29%
**TAS**	1 year	127 ± 36.8	136 ± 33.3	7.09%
	3 year	142 ± 35	150 ± 35.2	5.63%
	4 year	149 ± 36.3	157 ± 35.5	5.37%

Teachers’ Methodological Deficiencies (TMD); Academic Over-Burden (AOB); Beliefs About Performances (BAP); Public Interventions (PI); Negative Social Environment (NSE); Exams (EX); Content Worthlessness (CW); Participation Difficulties (PD) and Total Academic Stress (TAS).

**Table 6:** Differences in perceived classroom climate in Time 1 (before lockdown declaration) and Time 2 (after lockdown) based on Sex.

		Time 1	Time 2	Percentage Change
**Perceived Classroom Climate**				
**RCC**	Females	26.7 ± 5.24	26.8 ± 4.96	0.37%
	Males	26.5 ± 5.49	27.5 ± 4.31	3.77%
**RCT**	Females	27.8 ± 6.67	28.4 ± 5.47	2.16%
	Males	28.8 ± 5.33	28.5 ± 5.66	−1.04%

Responsibility Climate generated by Classmates (RCC) and Responsibility Climate generated by the Teacher (RCT).

**Table 7:** Differences in perceived classroom climate in Time 1 (before lockdown declaration) and Time 2 (after lockdown) based on Year of Study.

		Time 1	Time 2	Percentage Change
**Perceived Classroom Climate**				
**RCC**	1 year	29.6 ± 3.81	28 ± 4.62	−5.41%
	3 year	26.5 ± 4.65	26.8 ± 4.42	1.13%
	4 year	24.2 ± 6.19	26.4 ± 5.47	9.09%
**RCT**	1 year	30.2 ± 4.49	29.6 ± 5.36	−1.99%
	3 year	28 ± 6.11	27.5 ± 5.52	−1.79%
	4 year	26.4 ± 7.58	29.1 ± 5.45	10.23%

Responsibility Climate generated by Classmates (RCC) and Responsibility Climate generated by the Teacher (RCT).

## Data Availability

The data are not publicly available due to reasons concerning privacy of the subjects and since it belongs to an ongoing project.
